# The impact of an integrated intervention program combining drug therapy with water, sanitation, and hygiene (WASH) education on reinfection with intestinal parasitic infections among the Karen hill tribe in northern Thailand

**DOI:** 10.1186/s13071-024-06611-z

**Published:** 2024-12-29

**Authors:** Woottichai Nachaiwieng, Sangob Sanit, Nattharinee Kongta, Jassada Saingamsook, Suwit Duangmano, Sakorn Pornprasert, Pradya Somboon, Jintana Yanola

**Affiliations:** 1 School of Health Science, Mae Fah Luang University, Chiang Rai, 57100 Thailand; 2 Biomedical Technology Research Group for Vulnerable Populations, Mae Fah Luang University, Chiang Rai, 57100 Thailand; 3 Department of Parasitology, Faculty of Medicine, Chiang Mai University, Chiang Mai, 50200 Thailand; 4 Department of Medical Technology, Faculty of Associated Medical Sciences, Chiang Mai University, Chiang Mai, 50200 Thailand

**Keywords:** Intestinal parasitic infections, Soil-transmitted helminths, Karen hill tribe, WASH education, Mass drug administration

## Abstract

**Background:**

Intestinal parasitic infections (IPIs) are a major health problem among the Karen hill tribe in Thailand. This study aimed to evaluate the effect of an integrated intervention program of drug therapy combined with water, sanitation, and hygiene (WASH) education on reinfection with IPIs among the Karen hill tribe in an endemic area of northern Thailand.

**Methods:**

A quasi-experimental study was conducted in two Karen villages, involving 691 residents, in Omkoi District, Chiang Mai Province; one village was designated as the intervention group and the other as the control group. Baseline information was collected regarding the infections and participants’ knowledge, attitudes, and practices (KAP) related to prevention and control of IPIs. Detection of benzimidazole resistance linked to the beta-tubulin gene mutation in soil-transmitted helminths (STH) was performed using polymerase chain reaction (PCR) amplification and DNA sequencing. Mass drug administration (MDA) with albendazole was applied to both groups. The intervention group received WASH education, whereas the control group did not. Follow-up assessments were conducted at 3 and 6 months.

**Results:**

Baseline data revealed a 36.0% (123/342) prevalence of IPIs in the intervention group and 36.8% (96/261) in the control group. The most common helminth and pathogenic protozoan were *Trichuris trichiura* and *Giardia lamblia*, respectively. No non-synonymous mutations in the beta-tubulin gene were found. Post-intervention at 3-month and 6-month follow-ups revealed that the prevalence of IPIs in the intervention group was significantly decreased to 23.6% (*P* = 0.002) and 23.1% (*P* = 0.002), and the prevalence of pathogenic IPIs was reduced from 9.4% to 3.9% (*P* = 0.013) and 2.4% (*P* = 0.002), respectively. In contrast, no significant changes in the prevalence of IPIs were observed in the control group. The intervention group showed significant improvements in KAP scores, which were significantly higher than those in the control group.

**Conclusions:**

MDA alone is not effective for controlling IPIs among the Karen people due to rapid reinfection related to behavioral factors and socioeconomic conditions. We demonstrated for the first time that integration of WASH education increased KAP scores and consequently significantly reduced IPI reinfection among the Karen hill tribe in northern Thailand.

**Graphical Abstract:**

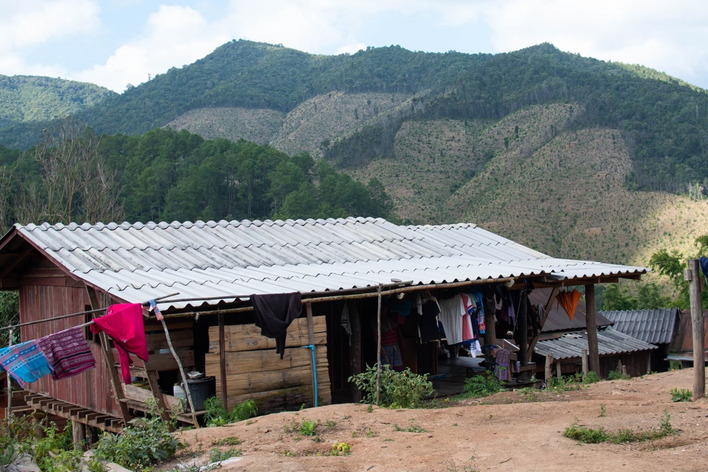

**Supplementary Information:**

The online version contains supplementary material available at 10.1186/s13071-024-06611-z.

## Background

Intestinal parasite infections (IPIs), mainly caused by soil-transmitted helminths (STH) and intestinal protozoa, are a major public health concern in tropical and subtropical climates, particularly among populations with limited resources [1, 2]. Infections with STH are recognized by the World Health Organization (WHO) as one of the neglected tropical diseases (NTDs), primarily affecting poor and undernourished people [3–6]. A high prevalence of IPIs is primarily related to low levels of education, lack of knowledge and awareness concerning the transmission and prevention of infections, restricted access to clean drinking water, poor hygiene practices, lack of sanitation, and pervasive food contamination [2]. Infection with IPIs is associated with anemia and malnutrition among other morbidities and chronic health and development problems.

Several preventive and control strategies including water, sanitation, and hygiene (WASH) intervention, community-led total sanitation (CLTS), and regular anthelminthic drugs, have been widely used against IPIs [2]. Mass drug administration (MDA) campaigns are essential for controlling STH infections, but their effectiveness is limited by high reinfection rates, and relying exclusively on them risks the emergence of drug resistance [7]. Integrating MDA with improved water, sanitation, and handwashing could effectively control STH transmission [8]. Drug treatment, water treatment, and increased sanitation are all used for controlling intestinal protozoa, specifically *Giardia lamblia* [9]. The evidence regarding the effectiveness of WASH interventions and WASH education in mitigating the risk of reinfection with IPIs is characterized by considerable variability [2, 10, 11]. Specifically, for STH, the available data present a mixed assessment of effectiveness [12]. A systematic review indicated that improved WASH practices can reduce the odds of STH infections by 33–70% [10]. Another review found that sanitation specifically reduced the odds of infections from *Ascaris lumbricoides* by 27%, *Trichuris trichiura* by 20%, and hookworm by 35% [13]. However, randomized controlled trials show mixed evidence regarding the effectiveness of WASH interventions on infection risk [12]. For example, the WASH Benefits trials in rural Kenya and Bangladesh evaluated the effects of WASH interventions on STH infections in children [14–16]. In Kenya, moderate-to-high WASH coverage resulted in a lower prevalence of *A. lumbricoides* with chlorine water treatment and in the combined WASH group [14, 15]. In Bangladesh, high uptake of WASH interventions led to reductions in *T. trichiura* prevalence in the sanitation group and lower hookworm prevalence in the chlorine treatment and combined WASH groups [16]. Additionally, the WASH for WORMS trial in Timor-Leste found no additional benefit of combining WASH with MDA for STH infection reduction compared to MDA alone [17, 18]. Conversely, a non-randomized study in Côte d’Ivoire indicated greater hookworm egg reduction rates when CLTS was combined with community-wide MDA compared to MDA alone [19]. Recently, a quasi-experiment study in children from Ethiopia revealed poor evidence of an additional benefit of improved WASH and health education on STH reinfection [20].

Thailand is a tropical country that has consistently reported numerous cases of IPI, particularly helminthiasis [21–23]. STH represent one of the two primary types of helminthiasis present, with hookworms constituting most of these cases. The national helminthic surveillance program in Thailand indicates a decline in the prevalence of STH, decreasing from 7.7% in 2009 [22] to 5.4% in 2019 [23]. Over recent decades, the prevalence of hookworm has also diminished; it was recorded at 11.4% in 2001 [21], decreasing to 6.5% in 2009 [22], and further to 4.5% in 2019 [23]. Historical national surveys in Thailand reveal that the prevalence of helminthiases varies by geographical region. The northeast region is considered an endemic area for *Opisthorchis viverrini*, while the southern region is endemic for hookworm [21–23]. In the northern region, the prevalence of helminth infection was reported at 9.7%, with a notably lower prevalence of 0.75% for STH recorded in the most recent national survey [23]. The high prevalence of IPIs among hill tribe people who live in the highlands with inadequate public utilities is a significant public health problem in Thailand [24, 25]. The prevalence of IPI in highland communities has ranged from 42.1% to 48.9% [25–27], surpassing the prevalence observed in impoverished lowland communities, which has varied from 5.8% to 37.3% [28–32]. This pattern has remained relatively stable in rural highland communities, particularly among economically disadvantaged hill tribe children in Chiang Mai Province, northern Thailand, since 1997 [26, 33]. Despite nationwide school-based deworming actions, 47.7% of Karen hill tribe children in Omkoi District, Chiang Mai Province had IPIs, with *T. trichiura* being the most common pathogenic parasite, followed by *A. lumbricoides* and *G. lamblia* [25]. This study aimed to evaluate the effect of an integrated program using MDA with anthelmintic drugs and WASH education on reinfection with intestinal helminthic and protozoa infections among Karen hill tribe people in an endemic area of Omkoi District, Chiang Mai Province, in northern Thailand.

## Methods

### Study design and population

**Fig. 1:**
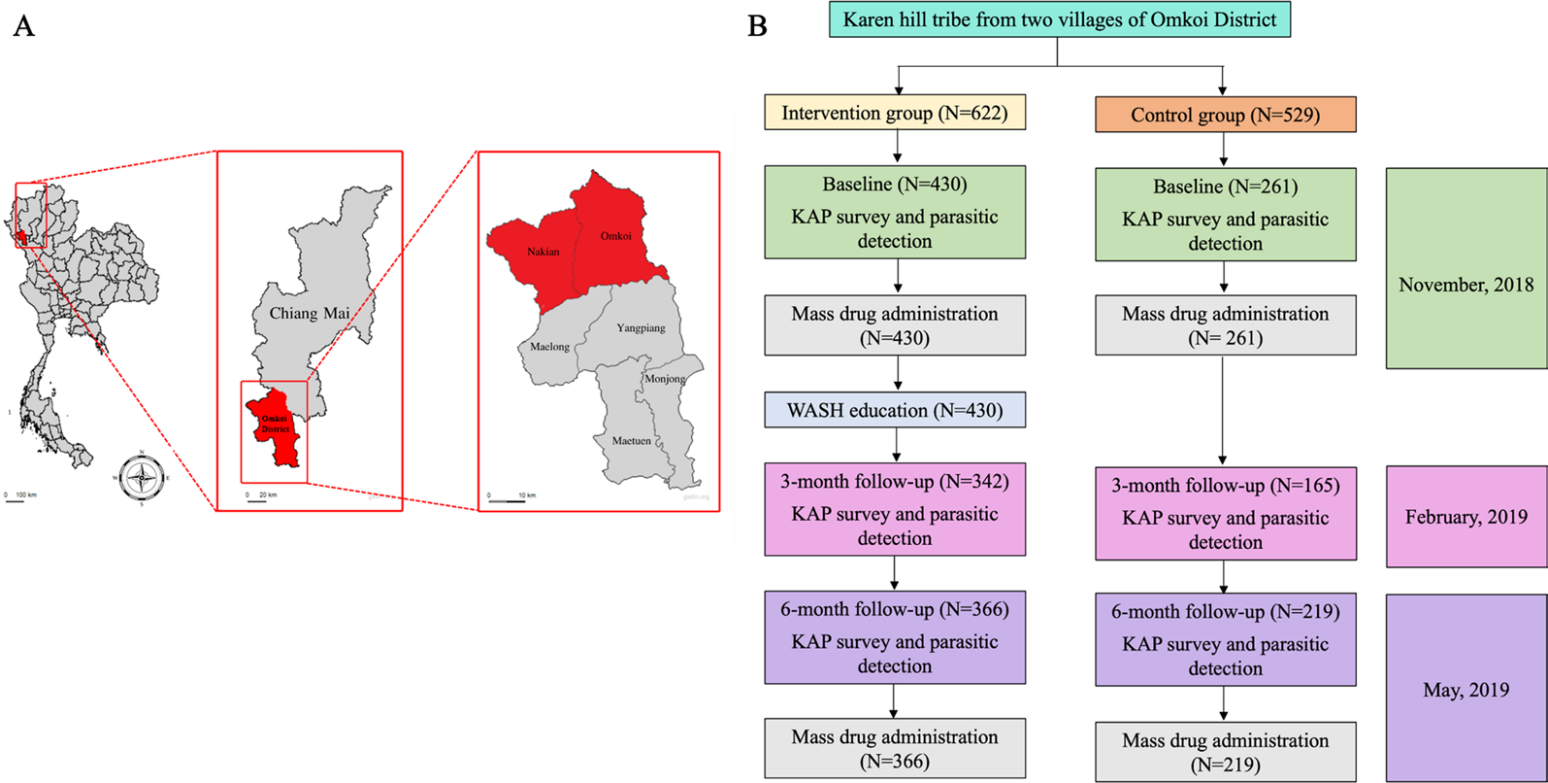
Study design and study area. **A**. Map of the study site. Omkoi District is situated southwest of Chiang Mai City in the Chiang Mai Province of the northern region of Thailand. The study areas consisted of two villages, with one village located in Omkoi subdistrict and the other in Nakian subdistrict. **B**. Study design

A quasi-experimental study was conducted between November 2018 and May 2019 in two rural Karen hill tribe villages in Chiang Mai Province, northern Thailand. The study area was in Omkoi District, about 187 km southwest of Chiang Mai city (Fig. 1A). The baseline situation in Omkoi District concerning the prevalence of IPIs and hygiene and sanitation conditions have been previously observed in Karen hill tribe schoolchildren with a high prevalence of IPIs [25]. A convenience sampling method was employed, meaning that participants were selected based on their availability and willingness to participate in this study. The two villages were allocated randomly, with one assigned to the intervention group and the other to the control group. The villages exhibited similar characteristics, including age, sex ratio, hygiene status, and village affiliation. All residents in the villages aged 6–81 years, except pregnant women, were invited to participate. The parameters to be assessed included intestinal helminth and protozoa prevalence, and participants’ knowledge, attitudes, and practices (KAP) related to IPIs and hygiene (Fig. 1B). An MDA for clearance of IPIs was provided to both groups (see details below). Only the intervention group received WASH education (Fig. 1B). The same fieldwork and laboratory procedures were repeated at 3 and 6 months after the collection of baseline data.

### Questionnaire construction

Sociodemographic data and participants' KAP information were gathered individually using a structured questionnaire administered by trained interviewers. The KAP questionnaire, designed by the authors following academic principles, comprised 10 questions for each domain. The knowledge domain questions assessed participants’ understanding of prevention and control of intestinal parasites, with multiple-choice questions featuring one correct answer among four options. Knowledge levels were categorized based on scores: high (score 8–10), moderate (score 6–7), and low (score below 5). The attitude domain questions explored opinions and thoughts about prevention and control of intestinal parasites, employing three-point Likert scales, where a score of 3 represented strong agreement and 1 denoted strong disagreement. Similarly, the practice domain questions, which assessed behaviors related to intestinal parasite prevention and control, used three-point Likert scales [34], with a score of 3 indicating the most frequent practice and 1 the least frequent. Attitude and practice domain levels were classified by scores: positive attitude or good practice (score 24–30), neutral attitude or moderate practice behavior (score 18–23), and negative attitude or poor practice (score below 17). The questionnaire's validity and reliability were assessed by three experts. Sociodemographic data and KAP assessments were conducted at baseline and 3 and 6 months after providing the intervention.

### Description of the intervention

**Fig. 2:**
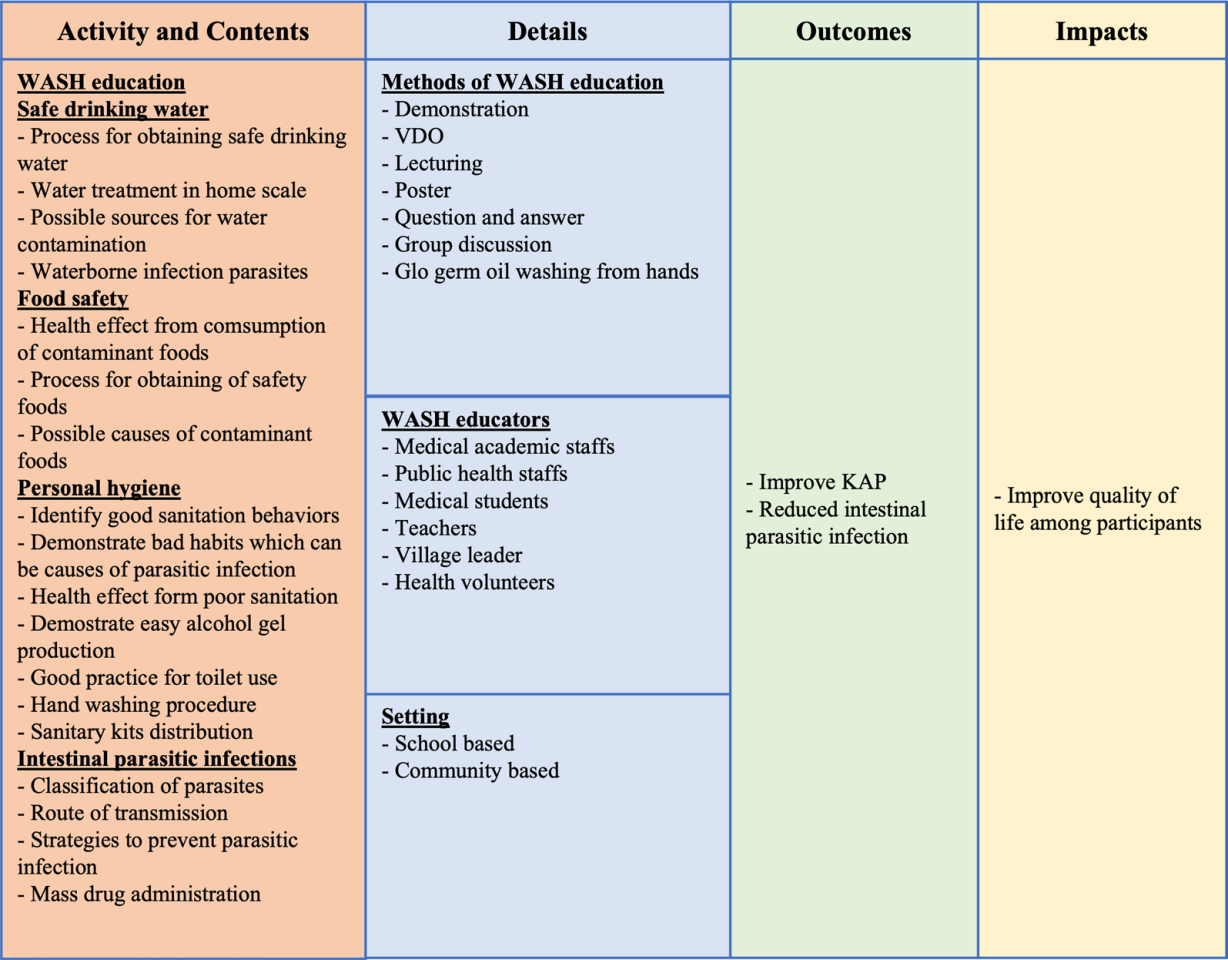
A framework showing the summary of the integrated intervention program in Omkoi District, Chiang Mai Province, northern Thailand

The intervention group received a range of activities and content which were overseen by medical staff (Fig. 2). The media used for knowledge implementation was a video clip (20 min) in Karen language relevant to school-based WASH education and community-based education, including parasitic infections in humans, safe drinking and food, good sanitation behaviors, and good practice in toilet use and hand-washing procedures using soap and water. The video clip was originally in Thai language and was translated to Karen language by academic Karen medical professionals with a strong understanding of both Thai and Karen languages. The video clip in Karen language was then re-translated back to the Thai language by another academic Karen medical professional for content approval by the editor. Regarding good practice for hand-washing procedures, Glo Germ oil (Glo Germ Company, USA) was lightly applied to participants’ hands and they were allowed to wash their hands using soap and water following WHO guidance [6]. Assessment of their hand-washing ability was performed after the training. Spoons, alcohol gel which would be used to wash hands only in the dry season, and hand towels were distributed to all participants to reduce the risk of parasitic contamination. In addition, village health volunteers and teachers were intensively trained in alcohol gel production and prevention and control strategies for IPIs. All participants received MDA with albendazole (Zenzera suspension, Bangkok drug company, Thailand) at a dosage of 400 mg, administered once daily for three consecutive days [35, 36], under the supervision of qualified clinicians at the Omkoi Hospital.

### Sample collection and intestinal parasite detection

The day before the stool collection, community health workers distributed clean and labeled plastic containers to all participants. They were invited to submit early-morning stool samples on the following day to a communal central place in the village. Stool samples contaminated with urine or water were rejected. Stool samples were transported in ice-boxes to the laboratory of the Department of Parasitology, Faculty of Medicine, Chiang Mai University, Thailand, and then analyzed immediately. Stool samples were examined by experienced medical technologists using a formalin ethyl acetate concentration method based on centrifugal sedimentation as described previously [25, 37]. In brief, approximately 2 g of an individual’s stool sample was stirred well in a 15-ml tube containing 10 ml of 10% formalin (Merck, Darmstadt, Germany) for fixation. The preserved fecal suspension was filtered through two layers of gauze into a centrifuge tube. Three milliliters of ethyl acetate (Merck, Darmstadt, Germany) was added, sealed with a stopper, and vigorously shaken and inverted for 30 sec for extraction of fat and debris. The tube was centrifuged at 500×*g* for 10 min. The debris plug and formalin-ether solution were discarded, and the sediment was resuspended. The entire suspension was examined under a microscope using 10× and 40× objectives, with iodine.

### Polymerase chain reaction (PCR) amplification and DNA sequencing of the beta-tubulin gene

To detect benzimidazole resistance associated with single-nucleotide polymorphisms (SNPs) in the beta-tubulin gene in *A. lumbricoides* and *T. trichiura*, positive stool samples at baseline collection were randomly selected for genomic DNA extraction. The parasite eggs were collected by a sugar floatation technique [38]. Genomic DNA was extracted from 100 pooled eggs per sample using a QIAamp Fast DNA stool mini kit (Qiagen, Hilden, Germany) according to the manufacturer’s instructions. The 564-base-pair (bp) and 472-bp fragments of the beta-tubulin gene for *A. lumbricoides* and *T. trichiura*, respectively, were amplified using PCR amplification (Additional file 1: Table S1) [39]. The PCR products were purified and sequenced in both directions by a commercial sequencing service (Macrogen, Seoul, Korea). The obtained Sanger sequences were subjected to sequence analyses using Geneious software (Biomatters, Auckland, New Zealand).

### Statistical analysis

The sociodemographic characteristics of all participants were summarized using descriptive statistics. Characteristics of the intervention and control group were compared using the Chi-square test. A paired *t*-test was used to assess the difference in KAP scores in both the intervention and control groups between the baseline and 3-month follow-ups and between the baseline and 6-month follow-ups, while the difference in individual KAP in the intervention group was analyzed by *t*-test. Differences in IPI prevalence in the intervention and control groups during the study were analyzed in each pair of the follow-up periods using the Chi-squared test.

## Results

### Sociodemographic data of participants

The intervention village had 622 residents, with 430 (69.1%) willing to participate. However, 342 and 366 participants were able to participate at the 3-month and 6-month follow-ups, respectively. In the intervention group (Table 1), approximately two-thirds of the participants were female. Most of them (70.2%) were aged between 6 and 15 years (the average age of the intervention group was 18 years). About 40% had a normal body mass index (BMI) (score 18.5–22.9). Over 75% of the participants had completed or were currently enrolled in basic and secondary education. Most participants were students with no salary; however, adult participants engaged in agricultural activities, mostly general employees with low income earning less than 5000 Thai Baht (approximately 140 US dollars) per month (Table 1). In the control village, there were 529 residents; 261 (49.3%) enrolled in this study, with an average age of 22 years. However, 165 and 219 were able to participate at the 3-month and 6-month follow-ups, respectively (Fig. 1B). Although there were minor variations in education, occupation, and salary, other factors exhibited no differences between the intervention village and the control village. These factors included sex, age, and BMI (Table 1).

**Table 1:** Sociodemographic characteristics of participants

Characteristics	Intervention group (*N* = 430)	Control group (*N* = 261)	*P*
	No. (%)	No. (%)	
Sex			
Male	151 (35.0)	102 (39.1)	0.278
Female	279 (64.5)	159 (60.9)	0.341
Age (years)			
6–15	302 (70.2)	165 (63.2)	0.056
16–25	29 (6.7)	20 (7.7)	0.619
26–35	35 (8.1)	23 (8.8)	0.747
36–45	37 (8.6)	23 (8.8)	0.927
46–55	20 (4.7)	13 (5.0)	0.858
≥ 56	7 (1.7)	17(6.5)	< 0.001***
BMI (kg/m^2^)			
< 18.5	173 (40.2)	99 (37.9)	0.548
18.5–22.9	170 (39.4)	114 (43.7)	0.265
23.0–24.9	29 (6.9)	22 (8.4)	0.467
25.0–29.9	42 (9.7)	19 (7.3)	0.280
≥ 30.0	16 (3.8)	7(2.7)	0.438
Language			
Karen	410 (95.3)	252 (96.6)	0.409
Thai	18 (4.2)	9 (3.4)	0.598
Shan	2 (0.5)	0 (0)	0.252
Education			
Illiterate	32 (7.4)	60 (23.0)	< 0.001***
Primary school	183 (42.6)	105 (40.3)	0.552
Secondary school	207 (48.1)	95 (36.4)	0.002**
Other	8 (1.9)	1 (0.4)	0.095
Sheltering			
With family	382 (88.8)	244 (93.5)	0.040*
Alone	39 (9.1)	16 (6.1)	0.158
Unspecified	9 (2.1)	1 (0.4)	0.070
Occupations			
Agriculturist	73 (17.0)	78 (29.9)	< 0.001***
General employee	34 (7.9)	2 (0.8)	< 0.001***
Entrepreneur	3 (0.7)	3 (1.1)	0.579
Undefined^a^	320 (74.4)	178 (68.2)	0.078
Salary (Thai baht/month)			
No income^a^	329 (76.5)	178 (68.2)	0.016*
< 5000	67 (15.6)	72 (27.6)	< 0.001***
5,000–10,000	22 (5.1)	11 (4.2)	0.590
10,001–15,000	11 (2.6)	1 (0.4)	0.033*
> 15,000	1 (0.2)	0 (0.0)	0.470

^a^Undefined occupation refers to students and unemployed persons. ^b^Most participants were students who did not specify their income. The differences observed between the intervention and control groups were marked at significance levels of *P < 0.05, **P < 0.01, and ***P < 0.001, tested using the Chi-square test

### KAP assessment of an integrated intervention program

The validity of the KAP questionnaire was evaluated by three experts on public health and parasitic infection. All questions in this questionnaire were found to be valid and reliable for assessing the efficiency of the program (Additional file 1: Table S2). The KAP assessments were conducted at 3- and 6-month follow-ups, and the results revealed a significant increase in KAP scores within the intervention group in all domains, in particular the knowledge score, which increased significantly from 5.04 to 5.70 (paired *t*-test, *P* < 0.001) after 3 months and from 4.89 to 5.46 (paired *t*-test, *P* < 0.001) after 6 months of follow-up. The attitude score increased significantly from 22.08 to 24.13 (paired *t*-test, *P* < 0.001) after 3 months, though it slightly decreased from 22.20 to 19.60 (paired *t*-test, *P* < 0.001) after 6 months of follow-up. The practice score increased significantly from 23.81 to 24.66 (paired *t*-test, *P* < 0.001) after 3 months and from 23.80 to 25.15 (paired *t*-test, *P* < 0.001) after 6 months of follow-up (Table 2). Interestingly, the attitudes progressed from a neutral to a positive level, while the practices improved from a moderate to a good level. In contrast, no improvements were observed in the control group. Furthermore, analysis of individual KAP within the intervention group revealed significant improvements in knowledge regarding infection routes and prevention methods (Additional file 1: Table S3) as well as in practices such as consuming thoroughly cooked food and practicing regular self-examinations (Additional file 1: Table S5). While attitudes demonstrated an initial increase, they subsequently declined. Consistent use of footwear, however, remained a persistent challenge (Additional file 1: Table S4).

**Table 2:** KAP assessment of the intervention and control groups at baseline and 3-month and 6-month follow-ups

	Intervention group						Control group					
	Comparison of KAP at baseline and 3-month follow-up (*N* = 342)			Comparison of KAP at baseline and 6-month follow-up (*N* = 366)			Comparison of KAP at baseline and 3-month follow-up (*N* = 165)			Comparison of KAP at baseline and 6-month follow-up (*N* = 219)		
	Baseline	3-month follow-up	*P*	Baseline	6-month follow-up	*P*	Baseline	3-month follow-up	*P*	Baseline	6-month follow-up	*P*
Knowledge (score mean ± SD)	5.04 ± 2.01	5.70 ± 2.17	< 0.001***	4.89 ± 1.99	5.46 ± 2.36	< 0.001***	4.26 ± 1.79	4.98 ± 1.92	< 0.001***	4.37 ± 1.85	4.96 ± 1.86	< 0.001***
High (score 8–10) [*n* (%)]	35 (10.2)	79 (23.1)	< 0.001***	32 (8.7)	82 (22.4)	< 0.001***	8 (4.8)	10 (6.1)	0.604	11(5.0)	21 (9.6)	0.065
Moderate (score 6–7) [*n* (%)]	109 (31.9)	110 (32.2)	0.933	109 (29.8)	102 (27.9)	0.571	36 (21.8)	26 (15.8)	0.164	55 (25.1)	54 (24.7)	0.923
Low (score 0–5) [*n* (%)]	198 (57.9)	153 (44.7)	0.001**	225 (61.5)	182 (49.7)	0.001**	121 (73.3)	129 (78.2)	0.300	153 (69.9)	144 (65.8)	0.359
Attitude (score mean ± SD)	22.08 ± 3.88	24.13 ± 3.79	< 0.001***	22.20 ± 3.78	19.60 ± 4.98	< 0.001***	22.32 ± 3.2	22.76 ± 4.58	0.283	22.53 ± 3.19	23.41 ± 3.47	0.002**
Positive (score 24–30) [*n* (%)]	140 (40.9)	201 (58.8)	< 0.001***	148 (40.4)	88 (24.0)	< 0.001***	57 (34.5)	67 (40.6)	0.253	82 (37.4)	112 (51.1)	0.004**
Neutral (score 18–23) [*n* (%)]	151 (44.2)	127 (37.1)	0.059	168 (45.9)	147 (40.2)	0.120	97 (58.8)	84 (50.9)	0.150	127 (58.0)	96 (43.8)	0.003**
Negative (score 0–17) [*n* (%)]	51 (14.9)	14 (4.1)	< 0.001***	50 (13.7)	131 (35.8)	< 0.001***	11 (6.7)	14 (8.5)	0.538	10 (4.6)	11 (5.0)	0.845
Practice (score mean ± SD)	23.81 ± 2.79	24.66 ± 2.76	< 0.001***	23.80 ± 2.83	25.15 ± 3.15	< 0.001***	24.78 ± 2.74	24.75 ± 4.76	0.949	24.87 ± 2.72	24.64 ± 3.81	0.388
Good (score 24–30) [*n* (%)]	194 (56.7)	233 (68.1)	0.002**	210 (57.4)	265 (72.4)	< 0.001***	122 (73.9)	121 (73.3)	0.902	161 (73.5)	149 (68.0)	0.206
Moderate (score 18–23) [*n* (%)]	140 (40.9)	109 (31.9)	0.015*	146 (39.9)	98 (26.8)	< 0.001***	40 (24.2)	40 (24.2)	1.000	55 (25.1)	64 (29.2)	0.335
Poor (score 0–17) [*n* (%)]	8 (2.3)	0 (0)	0.005**	10 (2.7)	3 (0.8)	0.050	3 (1.8)	4 (2.4)	0.704	3 (1.4)	6 (2.7)	0.338

The differences observed between baseline KAP assessments and subsequent follow-ups within each group are marked at significance levels of *P < 0.05, **P < 0.01, and ***P < 0.001, tested using paired *t*-tests

### Intestinal parasitic infections

A total of 603 Karen hill tribe people were enrolled at baseline for stool examination. No statistically significant differences in the overall prevalence of IPIs and species composition were observed in the intervention and control groups at baseline (Chi-square test, *χ*^2^ = 0.041, *df* = 1, *P* = 0.8394), with 36% infected with at least one parasite species (Table 3). Most stool specimens showed single infections (28.4%, 171/603), while double, triple, and quadruple infections were found in 7.1%, 0.7%, and 0.2%, respectively. Infection with STH was most common, with a prevalence of 6.3% (38/603), consisting of *T. trichiura* only (5.0%), *A. lumbricoides* only (0.8%), *T. trichiura* and *A. lumbricoides* (0.3%), and hookworm only (0.2%). The most prevalent intestinal protozoa were *G. lamblia* (2.2%, 13/603) and *Entamoeba coli* (28.8%, 174/603) for pathogenic and non-pathogenic protozoa, respectively.

**Table 3:** The intestinal parasite dynamics in the intervention and control groups during baseline and 3-month and 6-month follow-ups

Intestinal parasites	Intervention group, no. (%)			Control group, no. (%)		
	Baseline (*n* = 342)	3-month follow-up (*n* = 229)	6-month follow-up (*n* = 208)	Baseline (*n* = 261)	3-month follow-up (*n* = 165)	6-month follow-up (*n* = 219)
Overall IPIs	123 (36.0)	54 (23.6)^**^	48 (23.1)^**^	96 (36.8)	66 (40.0)	82 (37.4)
Single infection	92 (26.9)	48 (21.0)	42 (20.2)	79 (30.3)	51 (30.9)	66 (30.1)
Intestinal protozoa	84 (24.6)	45 (19.7)	41 (19.7)	60 (23.0)	41 (24.8)	49 (22.4)
*Entamoeba coli*	71 (20.8)	35 (15.3)	33 (15.9)	57 (21.8)	33 (20.0)	46 (21.0)
*Endolimax nana*	6 (1.8)	7 (3.1)	5 (2.4)	1 (0.4)	4 (2.4)	1 (0.5)
*Entamoeba histolytica/Entamoeba dispar*	1 (0.3)	1 (0.4)	NF	2 (0.8)	2 (1.2)	1 (0.5)
*Giardia lamblia*	5 (1.5)	2 (0.9)	2 (1.0)	NF	2 (1.2)	1 (0.5)
*Entamoeba hartmanni*	1 (0.3)	NF	1 (0.5)	NF	NF	NF
Intestinal helminths	8 (2.3)	3 (1.3)	1 (0.5)	19 (7.3)	10 (6.1)	17 (7.8)
*Ascaris lumbricoides*	2 (0.6)	NF	NF	2 (0.8)	NF	2 (0.9)
*Trichuris trichiura*	6 (1.8)	3 (1.3)	1 (0.5)	14 (5.4)	8 (4.8)	14 (6.4)
*Taenia* spp.	NF	NF	NF	2 (0.8)	1 (0.6)	1 (0.5)
*Enterobius vermicularis*	NF	NF	NF	1 (0.4)	1 (0.6)	NF
Polyparasitism	31 (9.1)	6 (2.6)^**^	6 (2.9)^**^	17 (6.5)	16 (9.0)	16 (7.3)
Intestinal protozoa	27 (7.9)	6 (2.6)^**^	6 (2.9)^*^	6 (2.3)	7 (4.2)	11 (5.0)
*E. coli* + *E. nana*	13 (3.8)	3 (1.3)	4 (1.9)	6 (2.3)	4 (2.4)	9 (4.1)
*E. coli* + *E. histolytica/E. dispar*	4 (1.2)	NF	NF	NF	3 (1.8)	2 (0.9)
*E. coli* + *G. lamblia*	6 (1.8)	2 (0.9)	1 (0.5)	NF	NF	NF
*E. nana* + *G. lamblia*	1 (0.3)	NF	1 (0.5)	NF	NF	NF
*E. coli* + *Sarcocystis hominis*	1 (0.3)	NF	NF	NF	NF	NF
*E. histolytica/E. dispar* + *E. nana* + *G. lamblia*	1 (0.3)	NF	NF	NF	NF	NF
*E. coli* + *E. nana* + *G. lamblia*	NF	1 (0.4)	NF	NF	NF	NF
*E. coli* + *E. histolytica/E. dispar* + *E. nana*	1 (0.3)	NF	NF	NF	NF	NF
Intestinal helminths and protozoa	4 (1.2)	NF	NF	11 (4.2)	8 (4.5)	5 (2.3)
*E. coli* + *T. trichiura*	2 (0.6)	NF	NF	8 (3.0)	2 (1.1)	1 (0.5)
*E. coli* + *A. lumbricoides*	NF	NF	NF	1 (0.4)	NF	NF
*E. coli* + *E. vermicularis*	NF	NF	NF	1 (0.4)	NF	NF
*E. coli* + *Taenia* spp.	NF	NF	NF	NF	2 (1.1)	NF
*E. coli* + hookworm	NF	NF	NF	NF	NF	1 (0.5)
*E. nana* + *T. trichiura*	NF	NF	NF	NF	NF	1 (0.5)
*E. histolytica/E. dispar* + *T. trichiura*	NF	NF	NF	NF	1 (0.6)	NF
*E. coli* + *A. lumbricoides* + *T. trichiura*	NF	NF	NF	1 (0.4)	1 (0.6)	NF
*E. coli* + *E. histolytica/E. dispar* + *T. trichiura*	NF	NF	NF	NF	1 (0.6)	NF
*E. coli* + *E. nana* + *E. vermicularis*	NF	NF	NF	NF	NF	NF
*E. coli* + *E. nana* + *T. trichiura*	NF	NF	NF	NF	NF	2 (0.9)
*E. coli* + *E. nana* + hookworm	1 (0.3)	NF	NF	NF	NF	NF
*E. coli* + *E. nana* + *T. trichiura* + *A. lumbricoides*	1 (0.3)	NF	NF	NF	NF	NF
*E. coli* + *E. histolytica/E. dispar* + *E. nana* + *T. trichiura*	NF	NF	NF	NF	1 (0.6)	NF

The differences observed between baseline assessments and subsequent follow-ups within each group are marked at significance levels of *P < 0.05 and **P < 0.01 tested using the Chi-square test. *NF* = not found

**Fig. 3:**
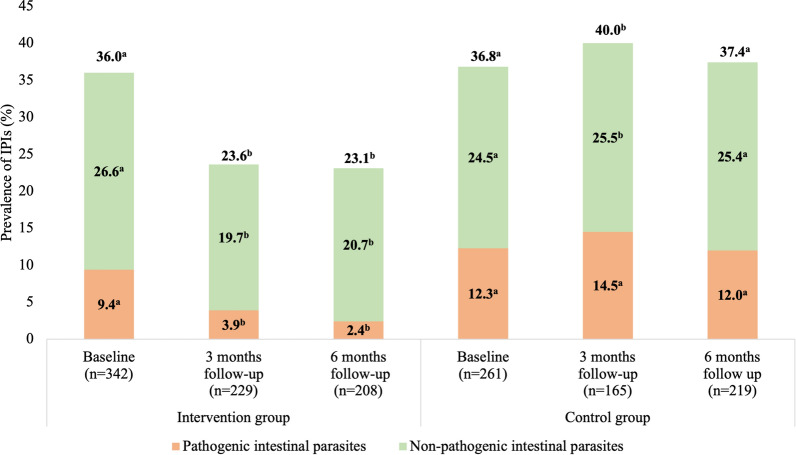
The prevalence of intestinal parasitic infections (IPIs) in the invention and control groups at baseline and 3-month and 6-month follow-ups. ^a,b^ represents a significant difference between baseline and follow-ups within each group at *P* < 0.05 tested using the Chi-squared test

In the intervention group, the overall prevalence of IPIs at baseline was 36.0% (123/342), of which 9.4% (32/342) harbored pathogenic intestinal parasites (Fig. 3 and Table 3). The prevalence of IPIs at the 3-month and 6-month follow-ups were significantly decreased to 23.6% (Chi-square test, *χ*^2^ = 9.84, *df* = 1, *P* = 0.0017) and 23.1% (Chi-square test, *χ*^2^ = 10.02, *df* = 1, *P* = 0.0015), respectively, constituting reductions of 34.4% and 35.8%. Interestingly, the pathogenic parasite infection prevalence was significantly reduced to 3.9% (Chi-square test, *χ*^2^ = 6.20, *df* = 1, *P* = 0.0128) and 2.4% (Chi-square test, *χ*^2^ = 10.05, *df* = 1, *P* = 0.0015), a reduction of 58.5% and 74.5%, at the 3-month and 6-month follow-ups, respectively. Conversely, no significant changes were observed in the control group.

The prevalence of *T. trichiura* infection in the intervention group was decreased from 2.6% to 1.3% (Chi-square test, *χ*^2^ = 1.14, *df* = 1, *P* = 0.2863) at the 3-month follow-up, and further decreased to 0.5% (Chi-square test, *χ*^2^ = 3.21, *df* = 1, *P* = 0.0732) at the 6-month follow-up (Table 3). The absolute reduction rates of *T. trichiura* infection were 50.0% and 80.8% at the 3-month and 6-month follow-up, respectively. Nine individuals were observed with *T. trichiura* eggs at baseline, but only two submitted their stool samples for both follow-up cycles and had no *T. trichiura* eggs. Correspondingly, the prevalence of *G. lamblia* in the intervention group was reduced from 3.8% to 2.2% (Chi-square test, *χ*^2^ = 1.15, *df* = 1, *P* = 0.2844) and 1.9% (Chi-square test, *χ*^2^ = 1.56, *df* = 1, *P* = 0.2116) at the 3-month and 6-month follow-ups, respectively, showing absolute reduction rates of 42.1% and 50.0% (Table 3). However, only 4 out of 13 individuals who tested positive for *G. lamblia* at baseline provided stool samples for subsequent follow-up cycles. Among the *G. lamblia*-positive cases, 50% did not exhibit *G. lamblia* cysts in the follow-up cycles, while others showed the presence of *G. lamblia* cysts. Notably, no *A. lumbricoides* or hookworm eggs were detected in the intervention group during the follow-up cycles. Moreover, during the 3-month and 6-month follow-up periods, there was a decrease in non-pathogenic protozoa prevalence in the intervention group from 26.6% to 19.7% (Chi-square test, *χ*^2^ = 3.591, *df* = 1, *P* = 0.0581) and 20.7% (Chi-square test, *χ*^2^ = 2.438, *df* = 1, *P* = 0.1184), respectively. In particular, the prevalence of *E. coli* decreased significantly from 33.0% to 17.9% (Chi-square test, *χ*^2^ = 15.86, *df* = 1, *P* < 0.001) and 18.3% (Chi-square test, *χ*^2^ = 14.01, *df* = 1, *P* < 0.001) at the 3-month and 6-month follow-up, respectively, showing an absolute reduction rate of 45.8% and 44.5%.

### Detection of benzimidazole resistance-associated SNPs in the beta-tubulin gene

Of the seven positive *A. lumbricoides* samples at baseline, five were successfully amplified and sequenced for the 564-bp fragment of the beta-tubulin gene (Additional file 1: Fig. S1). None of the known non-synonymous mutations in the beta-tubulin gene associated with benzimidazole resistance, including the SNP at codons 167, 198, and 200 [39–42], were found in a total of 500 tested eggs of *A. lumbricoides* (Additional file 1: Fig. S2). The heterozygous genotype of synonymous mutation at codon 179 (valine179), nucleotide change from GTG to GTA, was observed in two out of five positive samples. Unfortunately, none of the samples positive for *T. trichiura* were successfully amplified and sequenced for the 472-bp fragment of the beta-tubulin gene.

## Discussion

In this study, an integrated intervention program combining MDA with albendazole and WASH education was successful in reducing reinfection of IPIs among Karen hill tribe people in northern Thailand. The significant improvements across KAP scores in the intervention group compared to the control group highlight the effectiveness of the intervention in enhancing understanding, perceptions, and behaviors regarding the prevention and control of IPIs. MDA with anthelmintics alone was not effective in reducing the prevalence of IPIs because of reinfections related to poor knowledge of parasite transmission and control, and the poor hygiene and lifestyle of the Karen hill tribe. Our study is aligned with the implementation of WHO's 2020–2030 roadmap [6, 43]. We employed preventive measures to regulate the spread of pathogens in the environment, transitioning from managing morbidity to eliminating NTDs. Additionally, we utilized molecular platforms to investigate the resistance of specific STH to the current drugs of choice for their prevention.

At baseline, some Karen hill tribe people in both the intervention and control groups were infected with at least one intestinal parasite. The most common pathogen was *T. trichiura* for helminths and *G. lamblia* for protozoa. The composition and prevalence of the parasite species were similar to previous studies among school-age Karen hill tribe children [25, 26, 33]. All of these parasites except hookworm are transmitted to humans via the fecal-oral route. In the follow-up assessments, the integrated program led to significant reductions in the prevalence of intestinal parasites in the intervention group, particularly *A. lumbricoides*, hookworm, *T. trichiura*, *G. lamblia*, and non-pathogenic *E. coli*. However, there were instances of reinfection with *G. lamblia*. These findings suggest that the intervention measures employed were effective in decreasing the prevalence of the parasitic infections over time.

Many studies have been conducted on the recommended single dose of 400 mg by WHO or higher single dosages, which have shown that albendazole has limited efficacy against *T. trichiura* [44–46]. Some previous reports have indicated that the administration of three daily doses of albendazole (400 mg) for three consecutive days, as prescribed in this study, has resulted in increased efficacy against *T. trichiura* [47]. Recent studies have demonstrated that combined albendazole and ivermectin is more effective in treating trichuriasis than using either drug alone [48]. Albendazole has also been used for treating *G. lamblia*, especially in cases when metronidazole is ineffective [49]. However, albendazole has shown no efficacy against non-pathogenic protozoan *E. coli*. The proliferation of intestinal protozoa within the host can also have an impact on the overall intensity of the infection [50]. Moreover, various intestinal protozoa have an incubation period ranging from 7 to 28 days [51], indicating the possibility of reinfection occurring subsequent to treatment. The implementation of the WASH intervention is essential in mitigating the risk of reinfection, which can occur rapidly in the absence of preventive measures.

The emergence of benzimidazole resistance is a significant global concern, especially as WHO aims to control STH in high-risk populations by 2030 [6]. This goal relies primarily on large-scale deworming efforts using mebendazole or albendazole, both of which are derived from benzimidazole, for short-term control [52]. Notably, this preventive chemotherapy does not require individual diagnosis [52]. However, the emergence of resistance to benzimidazole could undermine these control strategies, particularly since such resistance is often seen in intestinal helminths of livestock that undergo routine deworming [53, 54]. The development of benzimidazole resistance in human STH is influenced primarily by treatment frequency [55–57]. More frequent administration of anthelmintics accelerates resistance emergence. Following treatment, surviving parasites gain a reproductive advantage over susceptible ones for approximately 2 to 3 weeks [55, 57]. Benzimidazole resistance is known to be related to mutations in the beta-tubulin gene at codons 167, 198, and 200 in several nematodes, including *A. lumbricoides* and *T. trichiura* [39–42]. However, none of the beta-tubulin gene mutations associated with benzimidazole resistance were found in our *A. lumbricoides*-positive samples at the baseline collection. *Ascaris lumbricoides* eggs were not found in the follow-up samples. Although the number of cases were low, the results suggest that our integrated program was effective against *A. lumbricoides* transmission in this Karen hill tribe community. Unfortunately, *T. trichiura*-positive samples could not be sequenced due to the lower yield of genomic DNA.

This study used the stool parasite concentration technique, formalin ethyl acetate, to detect the IPIs. The method is commonly used to identify a variety of intestinal parasites, both helminths and protozoa, and enables the widest range of parasite recovery with minimal susceptibility to technical errors [37, 58]. This study had limitations in monitoring the STH infections, as WHO advocates for the use of quantitative techniques such as Kato–Katz or other validated quantitative methods [6]. Although the egg production of STH may correlate with the overall worm burden, the quantity of eggs identified in a stool sample does not significantly influence anthelmintic drug therapy or subsequent follow-up treatment [37]. The process of helminth egg counting is inherently imprecise, influenced by variables such as the fecundity of the worms and the consistency of stool affected by dietary factors and transit times, as well as the expertise of the technician performing the analysis. Consequently, helminth egg counts do not provide a reliable estimate of the actual worm burden within the host [37].

IPIs are still a major public health concern in Thailand, particularly among ethnic groups with low socioeconomic status [25, 59, 60]. In this study, sociodemographic characteristics of Karen hill tribe participants in Omkoi District indicated low income within this population. Excluding the participants who had no salary, most of the participants in the intervention and control groups reported a monthly salary below 5000 THB. The latest current statistics indicate that 2304 individuals from a total population of 41,235 in Omkoi District were classified as poor [61]. Moreover, Omkoi District was classified as the poorest district in the country in 2020. The prevalence of IPIs is strongly associated with socioeconomic status, mediated by inadequate access to water and poor sanitation and hygiene [60]. There are various challenges to promoting positive change in poor communities that extend beyond provision of education. Karen people typically have low health education and an unsanitary lifestyle, which can lead to reinfection of intestinal parasites. This could be related to the ease with which these intestinal parasites are transmitted. During our survey, we observed that the toilets were used as storage rooms due to cultural beliefs that toilets should not be placed inside the house and also the lack of awareness about the consequences. The Karen hill tribe residing in Omkoi District in particular has a tradition of hand-grabbing rice for meals instead of using spoons.

WASH education has previously been used in areas of poor sanitation to reduce parasitic prevalence and improve participants’ sanitation behavior. However, the reduction rate of parasitic infection is still unsatisfactory [62]. Other studies have reported a strong capacity to implement a public health strategy that combines MDA, health education, and improvements in WASH education to control STH [12, 63]. With regard to economic factors in Omkoi area, our integrated intervention program offering basic sanitary items such as spoons, hand sanitizer, and hand towels could serve as a viable alternative besides MDA for mitigating the risk of IPIs transmitted through the fecal–oral route, and the results in reducing parasitic prevalence were highly satisfactory. Surprisingly, although our research area looks to be the most developed in Omkoi, with a nearly complete utility infrastructure, the rate of IPIs remains high. These might be due to a lack of personal sanitation concerns or their traditional eating behaviors. Therefore, this integrated intervention program could be applied to other less developed areas in Omkoi, where the utility systems are lacking or inadequate.

This study utilized the KAP questionnaire as a tool to evaluate the effectiveness of an intestinal parasitic reduction program based on WASH education, similar to prior research that tried to evaluate a health education learning package (HELP) to control STH infection using KAP and follow-up for 3 months [64]. Surprisingly, participants’ KAP scores increased significantly and persistently even 3 and 6 months after our program was implemented. This persistent increase indicates that the integrated intervention program successfully and effectively increases knowledge, improves attitudes, and promotes best practices in preventing parasite infections.

In addition, this study also investigated factors contributing to the KAP questionnaire to develop the integrated intervention program. In the domain of knowledge, participants displayed a lack of comprehension regarding the transmission of parasites through contaminated vegetables and the specimens used for parasitic investigation. As a result, a further integrated intervention program will increase the understanding of parasite contamination in vegetables by using multimedia and will provide good practices for vegetable washing. Consistent with a prior investigation [65] that highlighted the role of vegetable washing in mitigating the risk of parasitic infection, our findings align with and support this established correlation. Additionally, increasing the frequency of stool examinations may elevate individuals’ awareness concerning parasitic infections, as discussed in a previous report [66]. The observation of attitudes indicated a significant decline in positive attitude scores 6 months after implementation. This phenomenon highlights the need for constant and ongoing efforts in attitude development. As suggested in earlier health education programs, cultivating good attitudes, particularly among students, should be a monthly attempt, ideally arranged by educators [67]. Furthermore, a notable behavioral risk factor is the continued practice of not wearing footwear while walking on the parasite-contaminated ground. This includes not just wearing shoes but also addressing irregular shoe usage behaviors and the use of inappropriate protective footwear [68]. As a result, there is an urgent need to emphasize methods targeted at encouraging the use of suitable protective footwear when walking on parasite-contaminated ground.

Upon disseminating the final results to the intervention community, various stakeholders, including healthcare professionals, community leaders, teachers, and health volunteers, expressed apprehension regarding the prevalence of IPIs within the community. Consequently, they have formulated plans to execute certain components of the intervention program. These include the implementation of a handwashing campaign in schools, with a specific focus on school-age children as per the WHO guidelines for reducing STH [6]. Furthermore, they have proposed a plan to conduct annual stool examinations for the villagers and allocate budgets for the provision of clean drinking water, as well as MDA with anthelmintics within the community.

## Conclusions

This study demonstrates the effectiveness of an integrated intervention program aimed at decreasing IPIs among the Karen hill tribe residing in northern Thailand. The program, using MDA with albendazole and WASH education, successfully reduced the reinfection rate and improved the KAP in IPIs for prevention and control. The long-term effectiveness of the program is critical for sustained control and elimination of IPIs as a public health problem, but continuous support is required.

## Supplementary Information

Supplementary Material 1. Fig. S1 Agarose gel electrophoresis result of the 564-bp fragment of the A. lumbricoides beta-tubulin gene. Fig. S2 Multiple alignment of the *A. lumbricoides* beta-tubulin sequences. A reference sequence was use according to the sequence from Genbank number EU814697. Intron was highlight in gray color. Table S1 Primers and PCR amplification Protocol. Table S2 Validity and reliability of KAP questionnaire used in this study. Table S3 Assessment of knowledge questions within the KAP questionnaire after providing an integrated intervention program over 3 and 6 months compared to baseline in the intervention group. Table S4 Assessment of attitude questions within the KAP questionnaire after providing an integrated intervention program over 3 and 6 months compared to baseline in the intervention group. Table S5 Assessment of practice questions within the KAP questionnaire after providing an integrated intervention program over 3 and 6 months compared to baseline in the intervention group

## Data Availability

No datasets were generated or analyzed during the current study.
