# Supplementary material for: The impact of an integrated intervention program combining drug therapy with water, sanitation, and hygiene (WASH) education on reinfection with intestinal parasitic infections among the Karen hill tribe in northern Thailand
Source: Parasit Vectors. 2024 Dec 29;17:544. doi: 10.1186/s13071-024-06611-z (PMC11684068; doi:10.1186/s13071-024-06611-z)
Supplement: Supplementary file 1 — Supplementary Material 1. Fig. S1 Agarose gel electrophoresis result of the 564-bp fragment of the A. lumbricoides beta-tubulin gene. Fig. S2 Multiple alignment of the A. lumbricoides beta-tubulin sequences. A reference sequence was use according to the sequence from Genbank number EU814697. Intron was highlight in gray color. Table S1 Primers and PCR amplification Protocol. Table S2 Validity and reliability of KAP questionnaire used in this study. Table S3 Assessment of knowledge questions within the KAP questionnaire after providing an integrated intervention program over 3 and 6 months compared to baseline in the intervention group. Table S4 Assessment of attitude questions within the KAP questionnaire after providing an integrated intervention program over 3 and 6 months compared to baseline in the intervention group. Table S5 Assessment of practice questions within the KAP questionnaire after providing an integrated intervention program over 3 and 6 months compared to baseline in the intervention group [file 13071_2024_6611_MOESM1_ESM.docx]

**The integrated intervention program combining drug therapy with water, sanitation, and hygiene (WASH) significantly reduced the prevalence of intestinal parasitic infections among the Karen hill tribe in Northern Thailand**

**Woottichai Nachaiwieng^1,2^, Sangob Sanit^3^, Nattharinee Kongta^3^, Jassada Saingamsook^3^, Suwit Duangmano^4^, Sakorn Pornprasert^4^, Pradya Somboon^3^, Jintana Yanola^4*^**

1 School of Health Science, Mae Fah Luang University, Chiang Rai 57100, Thailand

2 Biomedical Technology Research Group for Vulnerable Populations, Mae Fah Luang University, Chiang Rai 57100 Thailand

3 Department of Parasitology, Faculty of Medicine, Chiang Mai University, Chiang Mai 50200, Thailand

4 Department of Medical Technology, Faculty of Associated Medical Sciences, Chiang Mai University, Chiang Mai 50200, Thailand

*Correspondence: [jintana.y@cmu.ac.th](mailto:jintana.y@cmu.ac.th)

**E-mail addresses**

WN: [woottichai.nac@mfu.ac.th](mailto:woottichai.nac@mfu.ac.th)

SS: [sangob.sanit@cmu.ac.th](mailto:sangob.sanit@cmu.ac.th)

NK: [nattharinee.k@cmu.ac.th](mailto:nattharinee.k@cmu.ac.th)

JS: [jassada.s@cmu.ac.th](mailto:jassada.s@cmu.ac.th)

SD: [suwit.du@cmu.ac.th](mailto:suwit.du@cmu.ac.th)

SP: [sakorn.pornprasert@cmu.ac.th](mailto:sakorn.pornprasert@cmu.ac.th)

PS: [pradya.somboon@cmu.ac.th](mailto:pradya.somboon@cmu.ac.th)

JY: [jintana.y@cmu.ac.th](mailto:jintana.y@cmu.ac.th)

**Supplementary information**

**
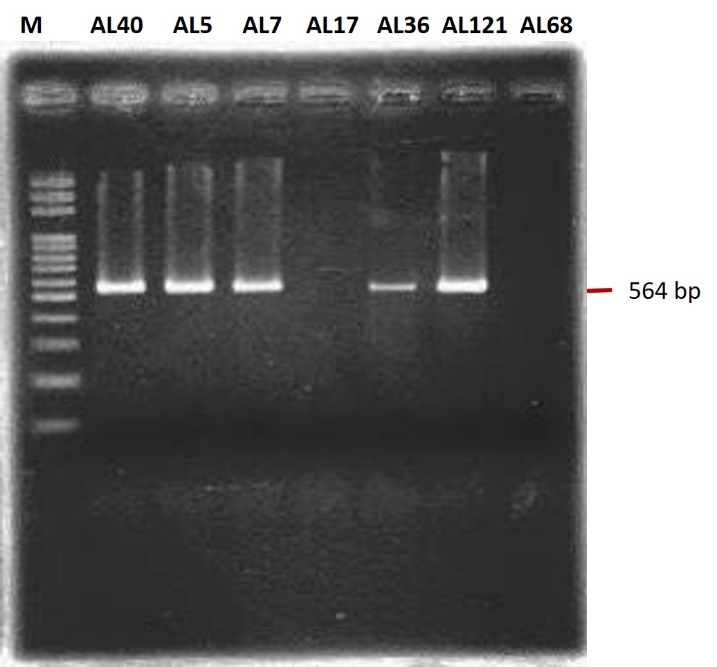
**

**Fig. S1** Agarose gel electrophoresis result of the 564-bp fragment of the *A. lumbricoides* beta-tubulin gene.


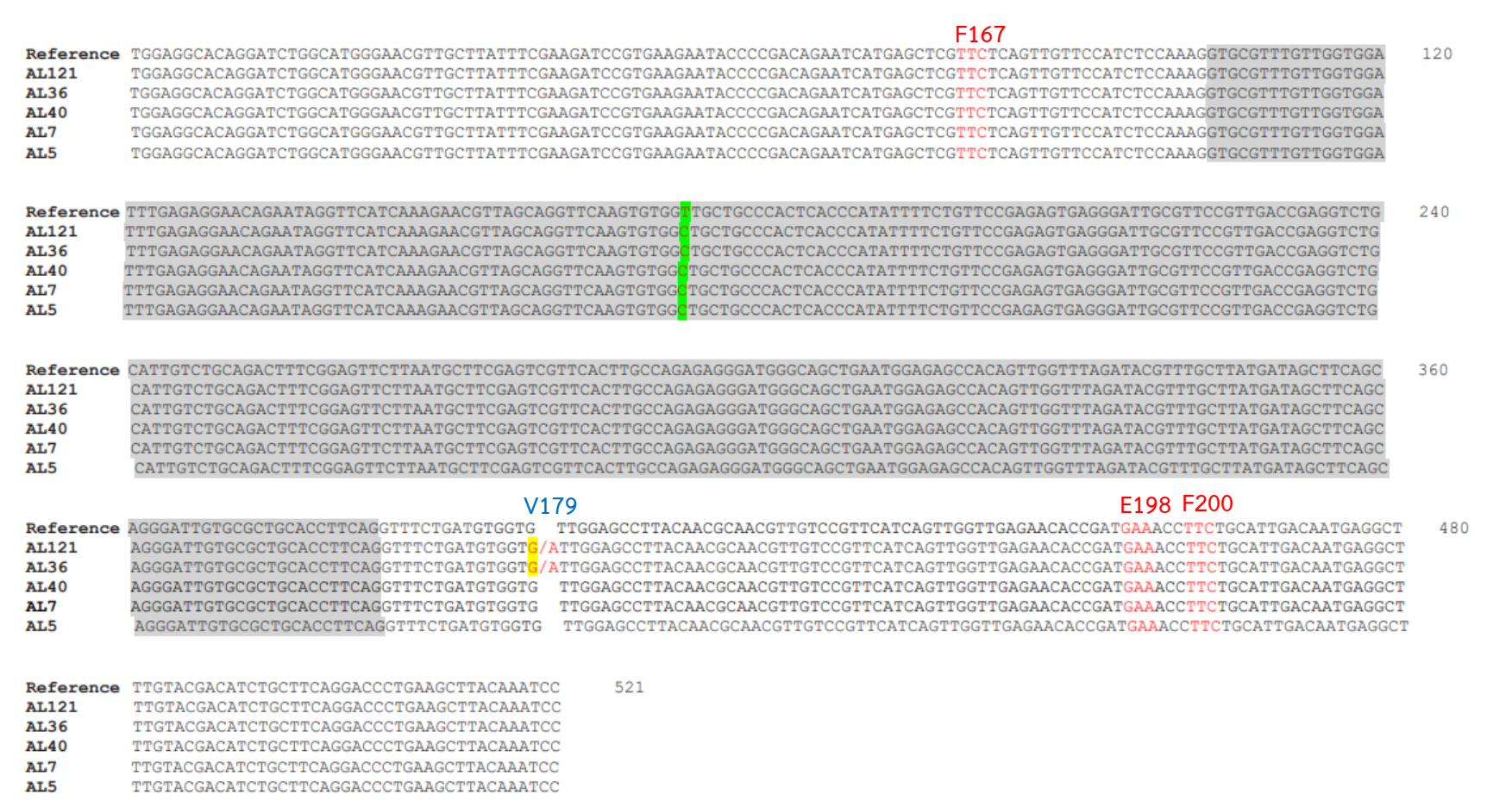


**Fig. S2** Multiple alignment of the *A. lumbricodes* beta-tubulin sequences. A reference sequence was use according to the sequence from Genbank number EU814697. Intron was highlight in gray color.

**Table S1 Primers and PCR amplification Protocol**

| **PCR component** | **Final concentration** |
| --- | --- |
| High Fidelity PCR buffer | 1X |
| dNTPs | 0.5 mM |
| MgSO_4_ | 2.5 mM |
| Platinum *Tag* DNA Polymerase High Fidelity (Invitrogen, Carlsbad, CA, USA) | 1.25 units |
| Forward and reverse primers ^a^ | 0.5 µM of each |
| **PCR cycles**  The amplifications consisted of an initial heat activation step at 94 ^o^C for 90 s, followed by 35 cycles of 94 ^o^C for 45 s, 59 ^o^C for 45 s and 68 ^o^C for 60 s with a final extension step at 68 ^o^C for 10 min. | |
| **Agarose electrophoresis**  PCR products were analyzed by electrophoresis on 2% agarose gel (Invitrogen, Carlsbad, CA, USA) using a voltage of 120 v for 25 min and visualized under UV light by Ethidium bromide staining. | |

^a^The forward and reverse primers for *A. lumbricoides* (AL-Btl-F: 5’-CCAGCTGACGCACTCGCTTGG-3’; AL-Btl-R: 5’-ATGGTTGAGGTCTCCGTATGTG-3’) and *T. trichiura* (TT-Btl-F: 5’-GCTAAA GGGCACTATACG -3’; TT-Btl-R: 5’-GGAAAGCGTAGGCATGTCG-3’) were used as previously described (Rashwan et al., 2017).

**Table S2** Validity and reliability of KAP questionnaire used in this study.

| **Validity of questionnaire** | **IOC** | **Acceptable IOC value**  (Tongprasert et al., 2014) |
| --- | --- | --- |
| Socio-demographic characteristic  Knowledge  Attitude  Practice  Overall | 0.76  0.90  0.93  0.80  0.85 | > 0.5  > 0.5  > 0.5  > 0.5  > 0.5 |
| **Reliability of questionnaire** | **Cronbach’s alpha coefficient** | **Acceptable IOC value**  (Tongprasert et al., 2014) |
| KAP questionnaire | 0.745-0.782 | > 0.7 |

**Table S3** Assessment of knowledge questions within the KAP questionnaire after providing an integrated intervention program over three and six months compared to baseline in the intervention group

| **Knowledge** | **Baseline**  **(N =342)** | | **3-month follow-up**  **(N =342)** | | ***t*** | ***P*** | **Baseline**  **(N =366)** | | **6-month follow-up**  **(N =366)** | | *t* | ***P*** |
| --- | --- | --- | --- | --- | --- | --- | --- | --- | --- | --- | --- | --- |
|  | **Right**  **N (%)** | **Wrong**  **N (%)** | **Right**  **N (%)** | **Wrong**  **N (%)** |  |  | **Right**  **N (%)** | **Wrong**  **N (%)** | **Right**  **N (%)** | **Wrong**  **N (%)** |  |  |
| 1. How can parasites infect to us?  2. Which helminths are capable of being spread by skin penetration?  3. Which helminths are capable of being spread by consuming improperly washed vegetables?  4. What is the general symptoms of parasitic infection?  5. How to prevent hookworm infection?  6. How can parasites be eradicated from human body?  7. Which helminths can cause cholangiocarcinoma? 8. Which one is classified to be intestinal parasite?  9. What form of helminth larvae are the cysts observed in pork?  10. Which sample is often used for parasite ova detection? | 199 (58.2)  66 (19.3)  77 (22.5)  200 (58.5)  111 (32.5)  267 (78.1)  142 (41.5)  234 (68.4)  144 (42.1)  284 (83.0) | 143 (41.8)  276 (80.7)  265 (77.5)  142 (41.5)  231 (67.5)  75 (21.9)  200 (58.5)  108 (31.6)  198 (57.9)  58 (17.0) | 223 (65.2)  138 (40.4)  73 (21.4)  220 (64.3)  132 (38.6)  287 (83.9)  147 (43.0)  268 (78.4)  168 (49.1)  295 (86.3) | 119 (34.8)  204 (59.6)  269 (78.6)  122 (35.7)  210 (61.4)  55 (16.1)  195 (57.0)  74 (21.6)  174 (50.9)  47 (13.7) | 2.522  6.595  -0.388  1.951  1.827  2.278  0.438  3.258  1.826  1.239 | 0.012*  <0.001*  0.638  0.052  0.069  0.023*  0.665  0.001*  0.069  0.216 | 205 (56.0)  72 (19.7)  77 (21.0)  202 (55.2)  122 (33.3)  285 (77.9)  137 (37.4)  241 (65.9)  147 (40.2)  302 (82.5) | 161 (44.0)  294 (80.3)  289 (79.0)  164 (44.8)  244 (66.7)  81 (22.1)  229 (62.6)  125 (34.1)  219 (59.8)  64 (17.5) | 230 (62.8)  136 (37.2)  74 (20.2)  228 (62.3)  150 (41.0)  288 (78.7)  166(45.4)  264 (72.1)  157 (42.9)  299 (81.7) | 136 (37.2)  230 (62.8)  292 (79.8)  138 (37.7)  216 (59.0)  78 (21.3)  200(54.6)  102 (27.9)  209 (57.1)  67 (18.3) | 2.505  5.425  -0.287  2.409  2.417  0.298  2.299  2.018  0.780  -0.337 | 0.013*  <0.001*  0.774  0.016*  0.016*  0.766  0.022*  0.044*  0.436  0.736 |

^*^ represents the significant difference between baseline and follow-ups at *P* < 0.050 tested using *t*-test

**Table S4** Assessment of attitude questions within the KAP questionnaire after providing an integrated intervention program over three and six months compared to baseline in the intervention group

| **Attitude** | **Baseline**  **(N = 342)** | | | **3-month follow-up**  **(N = 342)** | | | ***t*** | ***P*** | **Baseline**  **(N = 366)** | | | **6-month follow-up**  **(N = 366)** | | | ***t*** | ***P*** |
| --- | --- | --- | --- | --- | --- | --- | --- | --- | --- | --- | --- | --- | --- | --- | --- | --- |
|  | **Agree**  **N (%)** | **Neither agree nor disagree**  **N (%)** | **Disagree**  **N (%)** | **Agree**  **N (%)** | **Neither agree nor disagree**  **N (%)** | **Disagree**  **N (%)** |  |  | **Agree**  **N (%)** | **Neither agree nor disagree**  **N (%)** | **Disagree**  **N (%)** | **Agree**  **N (%)** | **Neither agree nor disagree**  **N (%)** | **Disagree**  **N (%)** |  |  |
| 1. Do you think eating raw meat will help you get stronger? | 80 (23.4) | 71 (20.8) | 191 (55.8) | 29 (8.5) | 73 (21.3) | 240 (70.2) | -5.214 | <0.001* | 80 (21.9) | 79 (21.6) | 207 (56.5) | 148 (40.4) | 131 (35.8) | 87 (23.8) | 8.836 | <0.001* |
| 2. Do you think helminths in raw meat and fish can be killed by smoking or salinity? | 72 (21.1) | 195 (57.0) | 75 (21.9) | 54 (15.8) | 184 (53.8) | 104 (30.4) | -2.551 | 0.011* | 76 (20.8) | 211 (57.6) | 79 (21.6) | 79 (21.6) | 172 (47.0) | 115 (31.4) | -1.825 | 0.069 |
| 3. Do you believe that if you walk off the ground barefoot, certain helminths may enter your skin? | 237 (69.3) | 55 (16.1) | 50 (14.6) | 257 (75.1) | 45 (13.2) | 40 (11.7) | 1.737 | 0.083 | 255 (69.7) | 57 (15.6) | 54 (14.7) | 145 (39.6) | 61 (16.7) | 160 (43.7) | -9.622 | <0.001* |
| 4. Do you think fermented fish can be eaten without boiling? | 81 (23.7) | 153 (44.7) | 108 (31.6) | 36 (10.5) | 125 (36.6) | 181 (52.9) | -6.397 | <0.001* | 83 (22.7) | 170 (46.4) | 113 (30.9) | 112 (30.6) | 145 (39.6) | 109 (29.8) | 1.624 | 0.105 |
| 5. Do you think important vitamins are often lost during the washing process? | 87 (25.4) | 98 (28.7) | 157 (45.9) | 66 (19.3) | 91 (26.6) | 185 (54.1) | -2.523 | 0.012* | 96 (26.2) | 100 (27.3) | 170 (46.5) | 139 (38.0) | 108 (29.5) | 119 (32.5) | 4.174 | <0.001* |
| 6. Do you believe parasites can infect you if you are exposed to contaminate soil near the toilet? | 173 (50.6) | 109 (31.9) | 60 (17.5) | 203 (59.4) | 104 (30.4) | 35 (10.2) | 3.270 | 0.001* | 190 (51.9) | 118 (32.2) | 58 (15.9) | 94 (25.7) | 140 (38.2) | 132 (36.1) | -7.941 | <0.001* |
| 7. Do you believe defecating outside the toilet is more pleasant than defecating inside the toilet? | 91 (26.6) | 82 (24.0) | 169 (49.4) | 63 (18.4) | 82 (24.0) | 197 (57.6) | -2.669 | 0.008* | 90 (24.6) | 94 (25.7) | 182 (49.7) | 156 (42.6) | 96 (26.2) | 114 (31.2) | 6.064 | <0.001* |
| 8. Do you believe cooking food before eating was sufficient to avoid helminthiasis? It is not necessary to wash your hands before eating. | 102 (29.8) | 72 (21.1) | 168 (49.1) | 56 (16.4) | 95 (27.8) | 191 (55.8) | -3.539 | <0.001* | 99 (27.0) | 83 (22.7) | 184 (50.3) | 151 (41.3) | 124 (33.9) | 91 (24.8) | 6.860 | <0.001* |
| 9. Do you believe you can use huge doses of anthelminthic medications without restriction? | 85 (24.9) | 129 (37.7) | 128 (37.4) | 55 (16.1) | 124 (36.3) | 163 (47.6) | -3.706 | <0.001* | 93 (25.4) | 133 (36.3) | 140 (38.3) | 73 (20.0) | 170 (46.4) | 123 (33.6) | -0.144 | 0.886 |
| 10. Do you believe parasites may spread from one member in the family to another? | 98 (28.6) | 159 (46.5) | 85 (24.9) | 145 (42.4) | 148 (43.3) | 49 (14.3) | 4.843 | <0.001* | 100 (27.3) | 180 (49.2) | 86 (23.5) | 93 (25.4) | 188 (51.4) | 85 (23.2) | -0.311 | 0.756 |

^*^ represents the significant difference between baseline and follow-ups at *P* < 0.050 tested using *t*-test.

**Table S5** Assessment of practice questions within the KAP questionnaire after providing an integrated intervention program over three and six months compared to baseline in the intervention group

| **Practice** | **Baseline**  **(N =342)** | | | **3-month follow-up**  **(N =342)** | | | ***t*** | ***P*** | **Baseline**  **(N =366)** | | | **6-month follow-up**  **(N =366)** | | | ***t*** | ***P*** |
| --- | --- | --- | --- | --- | --- | --- | --- | --- | --- | --- | --- | --- | --- | --- | --- | --- |
|  | **Always**  **N (%)** | **Sometimes**  **N (%)** | **Never**  **N (%)** | **Always**  **N (%)** | **Sometimes**  **N (%)** | **Never**  **N (%)** |  |  | **Always**  **N (%)** | **Sometimes**  **N (%)** | **Never**  **N (%)** | **Always**  **N (%)** | **Sometimes**  **N (%)** | **Never**  **N (%)** |  |  |
| 1. You always defecate inside the toilet | 229 (67.0) | 101 (29.5) | 12 (3.5) | 222 (64.9) | 120 (35.1) | 0 (0.0) | 0.440 | 0.660 | 235 (64.2) | 118 (32.2) | 13 (3.6) | 262 (71.6) | 95 (26.0) | 9 (2.4) | 2.445 | 0.015* |
| 2. You always wear shoes when you walk off the ground. | 221 (64.6) | 115 (33.6) | 6 (1.8) | 198 (57.9) | 138 (40.4) | 6 (1.7) | -2.019 | 0.044* | 225 (61.5) | 134 (36.6) | 7 (1.9) | 223 (60.9) | 138 (37.7) | 5 (1.4) | 0.000 | 1.000 |
| 3. You always wash your hands before meal. | 208 (60.8) | 128 (37.4) | 6 (1.8) | 229 (67.0) | 108 (31.6) | 5 (1.4) | 2.052 | 0.041* | 220 (60.1) | 139 (38.0) | 7 (1.9) | 248 (67.8) | 111 (30.3) | 7 (1.9) | 2.454 | 0.015* |
| 4. You always wash your hands after defecation | 205 (59.9) | 110 (32.2) | 27 (7.9) | 201 (58.8) | 118 (34.5) | 23 (6.7) | 0.000 | 1.000 | 215 (58.7) | 120 (32.8) | 31 (8.5) | 233 (63.7) | 118 (32.2) | 15 (4.1) | 2.511 | 0.012* |
| 5. You always consume raw freshwater fish, shrimp, and crabs | 67 (19.6) | 155 (45.3) | 120 (35.1) | 29 (8.5) | 133 (38.9) | 180 (52.6) | -5.727 | <0.001* | 72 (19.7) | 157 (42.9) | 137 (37.4) | 24 (6.6) | 153 (41.8) | 189 (51.6) | -5.577 | <0.001* |
| 6. You always consume raw pork, raw wild animal meat, or raw fermented pork | 62 (18.1) | 180 (52.6) | 100 (29.3) | 18 (5.23) | 167 (48.8) | 157 (45.9) | -6.241 | <0.001* | 66 (18.0) | 187 (51.1) | 113 (30.9) | 37 (10.1) | 159 (43.4) | 170 (46.5) | -5.028 | <0.001* |
| 7. You always thoroughly wash vegetables and fruits before consuming | 204 (59.6) | 123 (36.0) | 15 (4.4) | 213 (62.3) | 120 (35.1) | 9 (2.6) | 1.122 | 0.263 | 222 (60.7) | 129 (35.2) | 15 (4.1) | 238 (65.0) | 110 (30.1) | 18 (4.9) | 0.961 | 0.337 |
| 8. You always trim your nails | 173 (50.6) | 153 (44.7) | 16 (4.7) | 178 (52.1) | 158 (46.2) | 6 (1.7) | 1.247 | 0.213 | 177 (48.3) | 173 (47.3) | 16 (4.4) | 211 (57.6) | 147 (40.2) | 8 (2.2) | 3.345 | 0.001* |
| 9. You always investigate your stool for parasites at least once a year | 114 (33.3) | 134 (39.2) | 94 (27.5) | 159 (46.5) | 151 (44.1) | 32 (9.4) | 7.602 | <0.001* | 131 (35.8) | 142 (38.8) | 93 (25.4) | 208 (56.8) | 128 (35.0) | 30 (8.2) | 8.093 | <0.001* |
| 10. You always in contact with soil and sand | 62 (28.1) | 184 (53.8) | 96 (28.1) | 86 (25.1) | 184 (53.8) | 72 (21.1) | 2.761 | 0.006* | 62 (17.0) | 208 (56.8) | 96 (26.2) | 70 (19.1) | 188 (51.4) | 108 (29.5) | -0.215 | 0.830 |

^*^ represents the significant difference between baseline and follow-ups at *P* < 0.050 tested using *t*-test.

**References**

Rashwan, N., Scott, M., Prichard, R., 2017. Rapid genotyping of β-tubulin polymorphisms in Trichuris trichiura and Ascaris lumbricoides. PLoS Negl. Trop. Dis. 11, e0005205.

Tongprasert, S., Rapipong, J., Buntragulpoontawee, M., 2014. The cross-cultural adaptation of the DASH questionnaire in Thai (DASH-TH). J. Hand Ther. 27, 49–54. https://doi.org/10.1016/j.jht.2013.08.020
